# A comprehensive analysis of *Ardisia crenata* Sims from endophytes and rhizosphere soil microorganisms

**DOI:** 10.3389/fmicb.2025.1570230

**Published:** 2025-03-21

**Authors:** Chang Liu, Jiangli Luo, Demei Yang, Xiongwei Liu, Sixuan Zhou, Ying Zhou

**Affiliations:** ^1^School of Pharmacy, Guizhou University of Traditional Chinese Medicine, Guiyang, China; ^2^Guizhou Key Laboratory of Modern Traditional Chinese Medicine Creation, Guiyang, China; ^3^Institute of Animal Husbandry and Veterinary Sciences of Guizhou Province, Guiyang, China

**Keywords:** *Ardisia crenata* Sims, rhizosphere soil, endophytic, microorganisms, active compounds

## Abstract

**Introduction:**

Endophytic and rhizosphere microorganisms play crucial roles in influencing the quality and secondary metabolite accumulation of traditional Chinese medicinal.

**Methods:**

Endophytic and rhizosphere microorganisms play crucial roles in influencing the quality and secondary metabolite accumulation of traditional Chinese medicinal.

**Results and discussion:**

A total of 8,514,557 highquality reads were generated from 140 plant and soil sample in *A. crenata* Sims based on high-throughput sequencing. The fungal species composition within the endophytic and rhizosphere soil samples of *A. crenata* Sims is rich and varied, exhibiting notable disparities across different geographical regions of the plant. The alpha diversity and beta diversity indicated significant differences in microbial diversity and community structure between soil and plants. As for endophytic fungi, the dominant phyla in both plants and soil were Ascomycota and Basidiomycota, with different dominant genera between the two compartments. LEfSe analysis at the genus level identified 80 and 124 fungal indicator taxa associated with plants and soil, respectively, including *Aspergillus*, *Acremonium*, *Fusarium*, among others. Co-occurrence network analysis demonstrated intimate interactions among soil fungal microorganisms. Examination of soil physicochemical factors and the primary active constituent (bergenin) across different regions of *A. crenata* Sims indicated that the highest bergenin concentration is found in the Guangxi region, whereas the Guizhou region boasts relatively abundant soil nutrient components. Correlation analysis revealed that *Aspergillus*, *Fusarium*, *Penicillium*, *Tausonia*, and *Trichoderma* are correlated with soil physicochemistry or active compounds. These findings hint at a potential role for endophytic and rhizosphere microorganisms in the accumulation of active compounds within medicinal plants, thereby furnishing a scientific rationale for guiding the cultivation practices of *A. crenata* Sims.

## 1 Introduction

*Ardisia crenata* Sims, commonly known as zhu sha gen, refers to the dried root of the *Ardisia crenata* Sims plant, and is recognized as a special ethnic medicine in China (Liu et al., 2023). This species is predominantly found in regions south of the Yangtze River, particularly in Guizhou, Hunan, Guangdong, and Guangxi (Hu et al., 2020). Traditionally, it is used to clear heat and detoxify, disperse blood stasis and relieve pain, dispel wind, and eliminate dampness. It is known as a beneficial remedy for the throat among the Miao people, containing high concentration of bergenin and rich in various bioactive substances such as saponins, flavonoids, and coumarins, which exhibit antimicrobial, antiviral, anti-inflammatory, and anti-tumor effects (Wu et al., 2025). *A. crenata* Sims has significant medicinal value and is used for its roots, however, stems and leaves it has been found to be effective (Zhao Y. et al., 2024; Zhao C. et al., 2024; Tao et al., 2024; Tao et al., 2022). The roots, stems, and leaves of *A. crenata* Sims have very similar chemical compositions, however, there are significant differences in their contents (Zhao Y. et al., 2024; Zhao C. et al., 2024). Two new lactones, named Ardisicreolides A–B (1–2), were isolated from the ethyl acetate fraction of 70% ethanol extracts of dried leaves from *A. crenata* Sims (Tao et al., 2024). The content and types of active ingredients directly affect the efficacy of *A. crenata* Sims and determine their quality. These differences may be influenced by multiple factors, including genetic background, environmental conditions, and the interaction between plants and microorganisms.

The interaction between plants and microorganisms (endophytic fungi and rhizosphere) may affect the synthesis and accumulation of active ingredients. Endophytic fungi, as significant symbiotic microorganisms within plants, have been shown to significantly influence the chemical composition of their host plants by producing secondary metabolites or regulating the metabolic pathways of the hosts or generate new active components directly (Zheng et al., 2016; Sarkar et al., 2021; Wen et al., 2022). Endophytic fungi can affect plant growth by regulating photosynthesis, nutrient absorption, phytohormone levels, and other mechanisms (Rodriguez et al., 2009). Endophytic fungi are widely distributed and are found in various plants around the world, such as *Taxus chinensis* var. Mairei (Xu et al., 2023), *Panax notoginseng* (Sun et al., 2024), *Ginkgo biloba* (He et al., 2020), *Houttuynia cordata Thunb* (Ye et al., 2021), *Rosa roxburghii* Tratt (Zhang et al., 2021), and so on. Within the micro-ecosystem created by medicinal plants and endophytic fungi, endophytic fungi not only foster the growth of their hosts and enhance the host plants ability to withstand adverse environmental stress, but also promote the synthesis and accumulation of effective components within the host plants (Manzur et al., 2022; Ali et al., 2022; Toju et al., 2018). We also isolated and identified endophytic fungi from *A. crenata* Sims using traditional methods, finding a rich diversity that has sparked interest. The distribution of endophytic fungi varies across plant tissues (roots, stems, and leaves), and may also result in variations in the distribution and content of chemical components, as different endophytic fungi possess different metabolic capacities. At the same time, the diversity, composition and function of fungal community were also affected by soil–plant compartment (Zahid et al., 2021). The plant proactively recruits microbes by releasing exudates from its roots (Bulgarelli et al., 2012), these microbes can be further transferred to stems, and leaves, changes in the rhizosphere soil fungal community affects herbal yield and quality.

Microorganisms in soil, including fungi, are involved not only in nutrient cycling and organic matter transformation but also modify soil habitats through a variety of biochemical and biophysical mechanisms (Jansson and Hofmockel, 2020; Zhao Y. et al., 2024; Zhao C. et al., 2024). Microorganisms found in the plant rhizosphere, known as rhizosphere microorganisms, are closely related to the growth and development of plants and exhibit a strong rhizosphere effect. Rhizosphere microorganisms can accelerate the conversion and storage of effective nutrients within the rhizosphere soil, promoting nutrient uptake by the plant root system, which in turn affects herb quality (Yang et al., 2016). In the rhizosphere, there exists a complex network of “plant–soil-microbe” interactions (Morganj et al., 2005). The studies have shown that Rhizosphere microorganisms directly impact the growth of medicinal plants and the accumulation of medicinal components by facilitating nutrient absorption, enhancing disease resistance, and regulating the synthesis of secondary metabolites, such as, *Scutellaria baicalensis* (Dong et al., 2024). We have discovered that *A. crenata* Sims primarily grows in subtropical monsoon climate regions at altitudes ranging from 1,000 to 2,000 meters. It prefers to grow in moist forest underbrush and often coexists with pine needles. However, the chemical composition of *A. crenata* Sims varies depending on its origin. Does the rhizosphere microflora influence the accumulation of these compounds? Research into the relationships between rhizosphere soil microorganisms and the quality of herbs from *A. crenata* Sims has further clarified the synergistic relationships among plants, soil, and microorganisms within soil ecosystems.

Based on the above, we hypothesize that: the composition of endophytic and rhizosphere fungal communities in the *A. crenata* Sims from various origins exhibits geographical variations, and the different chemical compositions of *A. crenata* Sims in various regions may be related to endophytic fungi. Thus, in this study, high-throughput sequencing technology was utilized to investigate the community composition of endophytic and rhizosphere soil fungi associated with *A. crenata* Sims across seven regions in China, to elucidate the spatial dynamics of diversity and the relationships between endophytic and rhizosphere soil microorganisms. This study analyzed the correlation between soil factors, active compounds, and rhizosphere soil microorganisms to provide theoretical support for the artificial cultivation of *A. crenata* Sims and the standardization of planting bases. It also aimed to provide a scientific foundation for developing *A. crenata* Sims varieties with unique traits suitable for specific production areas.

## 2 Materials and methods

### 2.1 Sampling area description and sample collection

*Ardisia crenata* Sims samples were primarily collected between April and June 2024 from mountainous areas across seven distinct regions: Guiyang (GY), Bijie (BJ), Changshun (CS), and Duyun (DY) in Guizhou, as well as Huaihua in Hunan (HN), Qinzhou in Guangxi (GX), and Chaozhou in Guangdong(GD) (Table 1), and these areas all have a subtropical humid monsoon climate. The samples included whole healthy plants (without lesions) of *A. crenata* Sims and rhizospheric soil collected from the seven above-mentioned regions. Utilizing the five-point random sampling method, healthy and disease-free 1-2-year-old medicinal rhizomes of *A. crenata* Sims were selected from the seven mentioned regions, with the plants height ranging from 15–25 cm. Specifically, five plants, including leaves, stems, and roots, and corresponding five rhizosphere soil samples were collected from each region and transported to the laboratory in ice boxes.

**Table 1:** Geographical location and the meteorological data.

Region	Description
Guiyang	Located in central Guizhou, spans longitudes 106.07°E to 107.17°E and latitudes 26.11°N to 27.22°N, with an average altitude of 1,100 m. It has a vegetation coverage rate of 55–60%, annual precipitation of around 1,200 mm, and an average annual temperature of 15.3°C.
Bijie	Located in northwest Guizhou Province, covers longitudes 103.36°E to 106.43°E and latitudes 26.21°N to 27.46°N, with an average elevation of about 1,500 m, vegetation coverage of 60–65%, annual precipitation of 1,000–1,200 mm, and an average temperature of 13°C-15°C.
Changshun	Situated in central Guizhou, spans longitudes 106.11°E to 106.38°E and latitudes 25.38°N to 26.17°N, with average elevation of around 1,100 m, vegetation coverage of about 60%, annual precipitation of approximately 1,000 mm, and an average annual temperature of about 15°C.
Duyun	Located in the south of Guizhou Province, with longitude ranging from 107.07°E to 107.47°E and latitude ranging from 25.92°N to 26.38°N, with average elevation of around 800 m, vegetation coverage of about 65%, annual precipitation of approximately 1,200 mm, and an average annual temperature of about 16°C.
Huaihua	Situated in western Hunan, spanning longitudes 109.45°E to 111.06°E and latitudes 26.52°N to 28.29°N. It has an average altitude of 300 meters, vegetation coverage of about 70%, average annual precipitation of 1,300 millimeters, and an average temperature of 16°C.
Qinzhou	Situated in southern Guangxi, spans longitudes 108.41°E to 109.56°E and latitudes 21.35°N to 22.41°N. It has an average altitude of around 50 meters, vegetation coverage of about 60%, annual precipitation of approximately 1,600 millimeters, and an average temperature of about 22°C.
Chaozhou	Situated in eastern Guangdong, spans longitudes 116.22°E to 117.11°E and latitudes 23.26°N to 24.14°N. It has an average altitude of around 20 meters, vegetation coverage of about 60%, annual precipitation of approximately 1,600 millimeters, and an average temperature of about 22°C.

### 2.2 Sample processing

During the process of collecting rhizosphere soil, the surface layer soil is removed, and the plant roots are located and cut using sterilized small iron shovels. After gently shaking off the soil from the roots, they are placed in sterile plastic bags. Information regarding the sample collection sites and groupings is provided in Table 2. Plant tissue samples from roots, stems, and leaves were randomly selected from various plant regions and placed on a sterile, super-clean workbench for surface sterilization treatment. The samples were washed with sterile water for 30 s, soaked in 75% ethanol for 2 min, followed by 5 min soak in 2.5% NaClO, a 30 s soak in 75% sterile ethanol, and then washed 3–5 times with sterile water. The disinfected root, stem, and leaf tissues were then placed in a centrifuge tube and stored at −80°C, with five samples taken.

**Table 2:** General information of the collection sites in this study.

Collection site	Altitude (meters)	Longitude (E)	Latitude (N)	Plant group	Soil group
Guiyang city, Guizhou	1206.96	106.765326	26.537553	GYP	GYS
Bijie city, Guizhou	1511.00	105.2865	27.2858	BJP	BJS
Changshun country, Guizhou	1241.28	106.321433	26.119169	CSP	CSS
Duyun city, Guizhou	938.00	107.5157	26.3059	DYP	DYS
Qinzhou city, Guangxi	2141.50	104.2648	20.5409	GXP	GXS
Chaozhou city, Guangdong	1210.00	112.571143	22.262356	GDP	GDS
HuaiHua city, Hunan	1884.00	109.9500	27.5000	HNP	HNS

### 2.3 Determination of soil physiochemistry properties

The physical and chemical properties of the soil were determined using the following methods. pH value was tested through Water extraction-potentiometric method, Organic matter (OM) was examined by high temperature external thermal potassium dichromate oxidation capacity method, Total nitrogen (TN) was examined via Kjeldahl nitrogen determination method, Total phosphorus (TP) was determined via acid-soluble-molybdenum-antimony resistance colorimetry; Total Kalium (TK) was determined via hydrofluphysiochemistryoric acid-perchloric acid deboiling-flame spectrophotometry; Available nitrogen (AN) was determined via FeSO_4_-Zn reduction-alkaline hydrolysis diffusion method; Available phosphorus (AP) was determined via NaHCO_3_ method; Available Kalium (AK) was determined via ammonium acetate extraction-flame spectrophotometry. The canonical correspondence analysis (CCA) is a ranking method developed from correspondence analysis. It is primarily utilized to elucidate the relationship between environmental factors, active components of *A. crenata* Sims, and soil rhizosphere microbial fungal communities.

### 2.4 Detection of bergenin content in *Ardisia crenata* Sims

Accurately weigh approximately 200 mg sample into a stoppered conical flask. Precisely add 20 mL of methanol, and record the total weight. Sonicate the mixture for 40 min, then cool to room temperature. Re-weigh the flask and replenish any weight loss with methanol. Mix thoroughly and filter. Precisely transfer 5 mL of the subsequent filtrate to a 10 mL volumetric flask, dilute to volume with methanol, and mix well. Accurately weigh 10 mg of bergenin reference standard and transfer to a stoppered conical flask. Precisely add 10 mL of methanol to prepare a stock solution (1 mg/mL). Dilute an appropriate volume of the stock solution with methanol to obtain a working solution at 50 μg/mL. Chromatographic Conditions and System Suitability: Column: Octadecylsilane (ODS)-bonded silica gel (4.6 mm × 250 mm, 5 μm). Mobile phase: Methanol–water (25:75, v/v), isocratic elution. Flow rate: 1.0 mL/min. Detection wavelength: 275 nm. Injection volume: 5 μL. System suitability: Theoretical plate number (N) ≥ 5,000, tailing factor (T) ≤ 1.5, and resolution (Rs) ≥ 2.0 between bergenin and adjacent peaks. The 2020 edition of the Chinese Pharmacopoeia has established bergenin as the quality control index for *A. crenata* Sims, stipulating that its content should not be less than 1.5%. The quality of the *A. crenata* Sims herbs collected in this study was tested to meet the standards for medicinal use.

### 2.5 Sample DNA extraction, PCR amplification, and library sequencing

The plant tissues, including roots, stems, and leaves, along with the rhizosphere soil sample, were employed for the extraction of fungal genomic DNA. The genomic DNA extraction Kit (Solarbio, China) was utilized to extract DNA, following the manufacturer’s instructions. Upon completion of the genomic DNA extraction, the extracted material was analyzed using 1% agarose gel electrophoresis. The ITS1F (5’-CTTGGTCATTTAGAGGAAGTAA-3′) and ITS2R (5’-GCTGCGTTCTTCATCGATGC-3′) primers were used for amplifying fungal ITS regions, resulting in approximately 300 bp amplicon size (White et al., 1990). The fungal PCR reaction conditions were: 5 min at 95 C; 20 cycles of 95 C for 45 s, 57 C for 30 s, and 72 C for 30 s; and 72 C for 10 min. The amplified products were purifed and mixed in equivalent amounts. The PCR products was extracted from 2% agarose gel and purified using the PCR Clean-Up Kit (YuHua, Shanghai, China) according to manufacturer’s instructions and quantified using Qubit 4.0 (Thermo Fisher Scientific, USA). Purified amplicons were pooled in equimolar amounts and paired-end sequenced on an Illumina Nextseq2000 platform (Illumina, San Diego,USA) according to the standard protocols by Majorbio Bio-Pharm Technology Co. Ltd. (Shanghai, China). This data can be found here: PRJNA1231709.

### 2.6 Sequencing data processing

After removing the barcode and primer sequences, the FLASH software (Version 1.2.11) was used to assemble the reads into raw tags (Magoč and Salzberg, 2011). The resulting raw tags were subjected to quality control using fastQ software (Version 0.20.0) (Wingett and Andrews, 2018), resulting in high-quality clean reads. Subsequently, the Vsearch (Version 2.15.0) (Rognes et al., 2016) software was utilized to align clean tags with databases, for the detection and removal of chimeras, resulting in effective tags. QIIME2 software (Zhang et al., 2019) was utilized for Amplicon Sequence Variants (ASV) clustering analysis.

### 2.7 Bioinformatics analysis

To assess the variations in Alpha diversity indices across different groups, the Kruskal-Wallis test was employed to compare the differences between them. Should the Kruskal-Wallis test reveal significant differences among the groups, subsequent *post hoc* tests could be performed to identify which specific groups exhibit these differences, with *p*-values adjusted using the False Discovery Rate (FDR) to minimize false positives. Beta diversity analysis was conducted based on Unifrac distances, PCA and PCoA was employed to analyze the significance of differences in community structure between groups (Lutsiv et al., 2021). PCA was used to reduce the dimensionality of the environmental and community data, to identify the primary axes of variation influencing fungal and plant communities. PCoA was applied to assess differences in community composition based on beta diversity metrics, such as Bray–Curtis dissimilarity. The LEfSe (Chang et al., 2024) was used to perform differential species analysis between groups, with taxa with an LDA score > 3.0 and *p-*values less than 0.05 considered significantly different. A co-occurrence network analysis was conducted to illustrate microbial community relationships using the Spearman correlation index calculation.

### 2.8 Statistical analysis

The soil physiochemistry properties and active ingredient data are presented as mean ± SD (standard deviation of the mean) and analyzed using SPSS 26.0 software. For three or more groups, one-way analysis of variance (ANOVA, *p* < 0.05) was applied, with adjustments for multiple comparisons using Tukey’s Honest Significant Difference (HSD) or the Waller-Duncan test followed by Dunn’s multiple comparisons test to confirm statistically significant differences. The significant letter marking method was used to indicate significant differences among multiple sets of data. Different lowercase letters for the same indicator represent significant differences (*p* < 0.05), and the same lowercase letters represent non-significant differences (*p* > 0.05).

## 3 Results

### 3.1 Basic physicochemical properties of rhizosphere soil and active ingredient of *Ardisia crenata* Sims

**Figure 1:**
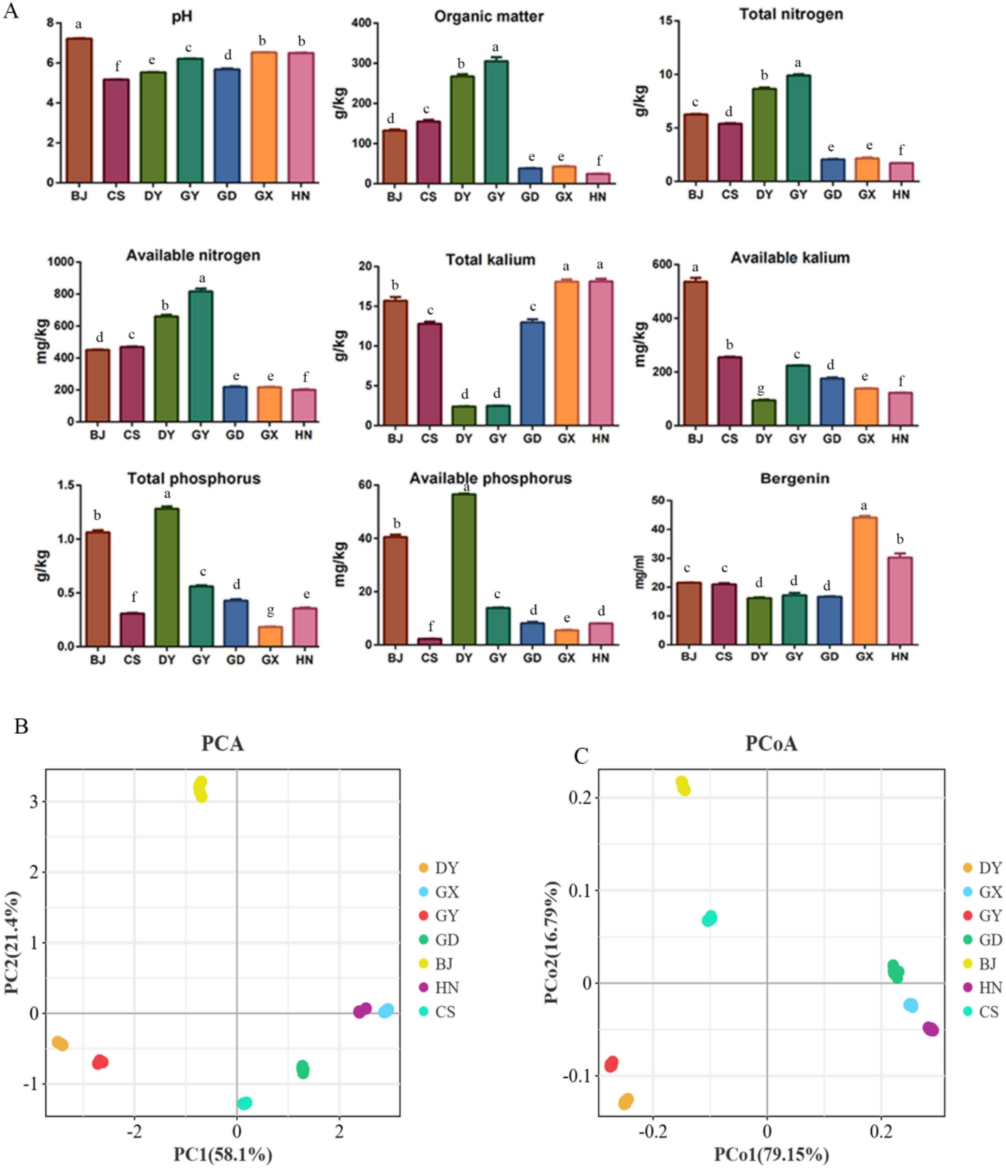
The physicochemical properties of rhizosphere soil and active ingredient of root from *Ardisia crenata* Sims in different producing areas. Guiyang: GY, Bijie: BJ, Changshun: CS, Duyun: DY, Hunan: HN, Guangxi: GX, and Guangdong: GD. The data are presented as the means ± SD (*n* = 3). Different lowercase letters for the same indicator represent significant differences (*p* < 0.05), and the same lowercase letters represent non-significant differences (*p* > 0.05). **(A)** physicochemical properties and active ingredient; **(B)** The PCA analysis; **(C)** The PCoA analysis.

The results indicated that the soil physiochemistry properties and active ingredient of *A. crenata* Sims rhizosphere soil varied among different region (Figure 1A). There was difference in the pH values of the soil samples from the 7 region, however, the soil pH value of Changshun (CS) was acidic, while that of Bijie (BJ) was alkaline. In general, the levels of Organic Matter (OM), Total Nitrogen (TN), Available Nitrogen (AN), Available Kalium (AK), Total Phosphorus (TP), and Available Phosphorus (AP) in the four regions of Guizhou were higher than those in Guangdong (GD), Guangxi (GX), and Hunan (HN) (*p* < 0.05), except for Total Kalium (TK). The contents of OM, TN and AN were the highest in Guiyang (GY) (*p* < 0.05), and the lowest in HN. The contents of TP and AP were the highest in Duyun (DY) (*p* < 0.05), and the contents of AK were the highest in BJ(p < 0.05). Among the seven sample plots, GX had the highest bergenin content, whereas GY had the lowest (Figure 1A) (*p* < 0.05). The PCA and PCoA analyses indicated that the soils from BJ, DY, GY, and CS belong to a single category, while the soils from GD, GX, and HN exhibit similarities (Figures 1B,C).

### 3.2 Analysis of endophytes and rhizosphere soil microorganisms diversity

**Figure 2:**
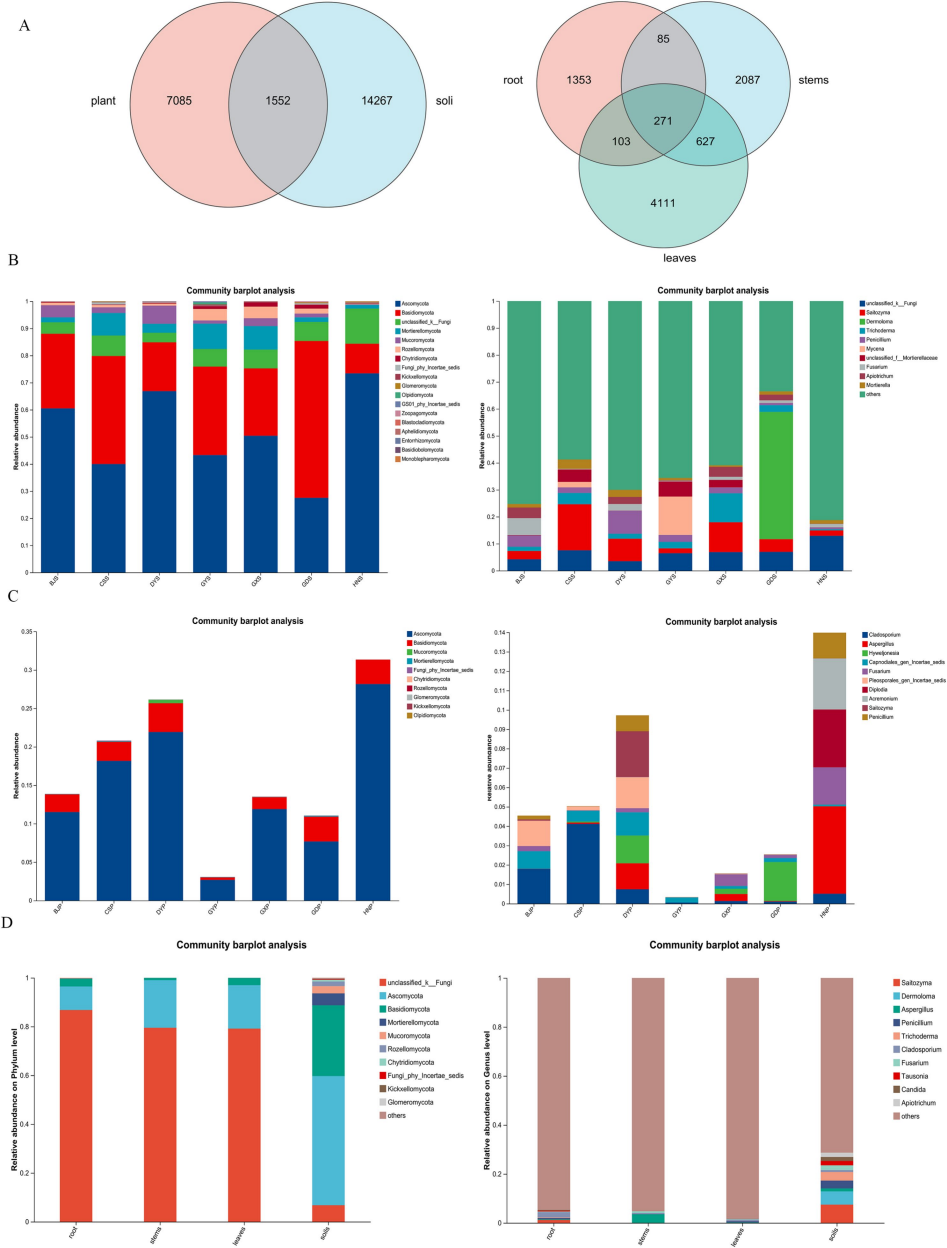
Composition and relative abundance of fungal community at phylum and generic level in rhizosphere soil and plant of *A. crenata* Sims in different producing areas. **(A)** Venn diagrams of fungal amplicon sequencing variants (ASVs) of plants and rhizosphere soil; **(B)** Relative abundance of fungi at the phylum level and genus level in soil; **(C)** Relative abundance of fungi at the phylum level and genus level in plants; **(D)** Relative abundance of fungi at the phylum level and genus level in leaves, stems, roots and soils.

Based on high-throughput sequencing, a total of 8,514,557 high-quality reads were generated from 140 plant and soil sample with an average of 347 bp. After valid sequences from every sample were grouped into Amplicon Sequence Variants (ASVs) with 97% concordance, species annotations were added to the representative sequences of each ASV. The number of ASVs varied significantly between plant and soil samples from each site, with soil samples more than plant samples. A few endophytic fungi were shared between the plants and soil (Figure 2A). Therefore, soil hosts a greater diversity of microbial species compared to plants, and variations in soil types can further influence the diversity of endophytic fungi within plants.

Based on ITS sequencing, Soil fungi, had an overall higher diversity across all taxa representing 18 phylum, 85 classes, 238 orders, 627 families and 1,618 genus. Studies have shown that the relative abundance of endophytic fungi varies across different regions of rhizosphere soil samples, with the Ascomycota, Basidiomycota, Mortierellomycota, Mucoromycota, and Rozellomycota being the dominant phyla (Figure 2B). The content of *Ascomycota* was higher in HNS and DYS samples, while the content of *Basidiomycota* was higher in GDS and CSS samples. In the Guizhou region, including BJS, CSS, DYS, and GYS, Mortierellomycota and Mucoromycota exhibited higher abundances. The relative abundance of endophytic fungi was depicted at different region of rhizosphere soil samples in genus level, and “Unclassified” was the predominant groups, *Saitozyma* and *Penicillium* were the main known genus in Guizhou region, including BJS, CSS, DYS, and GYS.

The fungal communities in *A. crenata* Sims belong to 11 phylum, 51 classes, 165 orders, 460 families, and 1,132 genus. In addition to significant differences in endophytic fungal communities across different rhizosphere soil samples, there was also an impact of *A. crenata* Sims plants from different regions. At phylum level, Ascomycota and Basidiomycota were the main known phylum (Figure 2C). The *Saitozyma*, *Dermoloma*, *Trichoderma*, *Penicillium*, *Fusarium* were the main known at the genus level (Figure 2C). The content of *Cladophialophora* was higher in CSP, BJP, and DYP samples, while the content of *Aspergillus* was higher in HNP samples. In conclusion, endophytic fungi demonstrated significant diversity and community changes across different plant and rhizosphere soil. There were significant differences in endophytic fungi between Hunan region (HN) and Guizhou region (DY, BJ, CS, and GY). A large number of unidentified endophytic fungi within the roots, stems, and leaves of plants (Figure 2D), a finding that warrants further research.

### 3.3 Analysis of microbial alpha diversity

**Figure 3:**
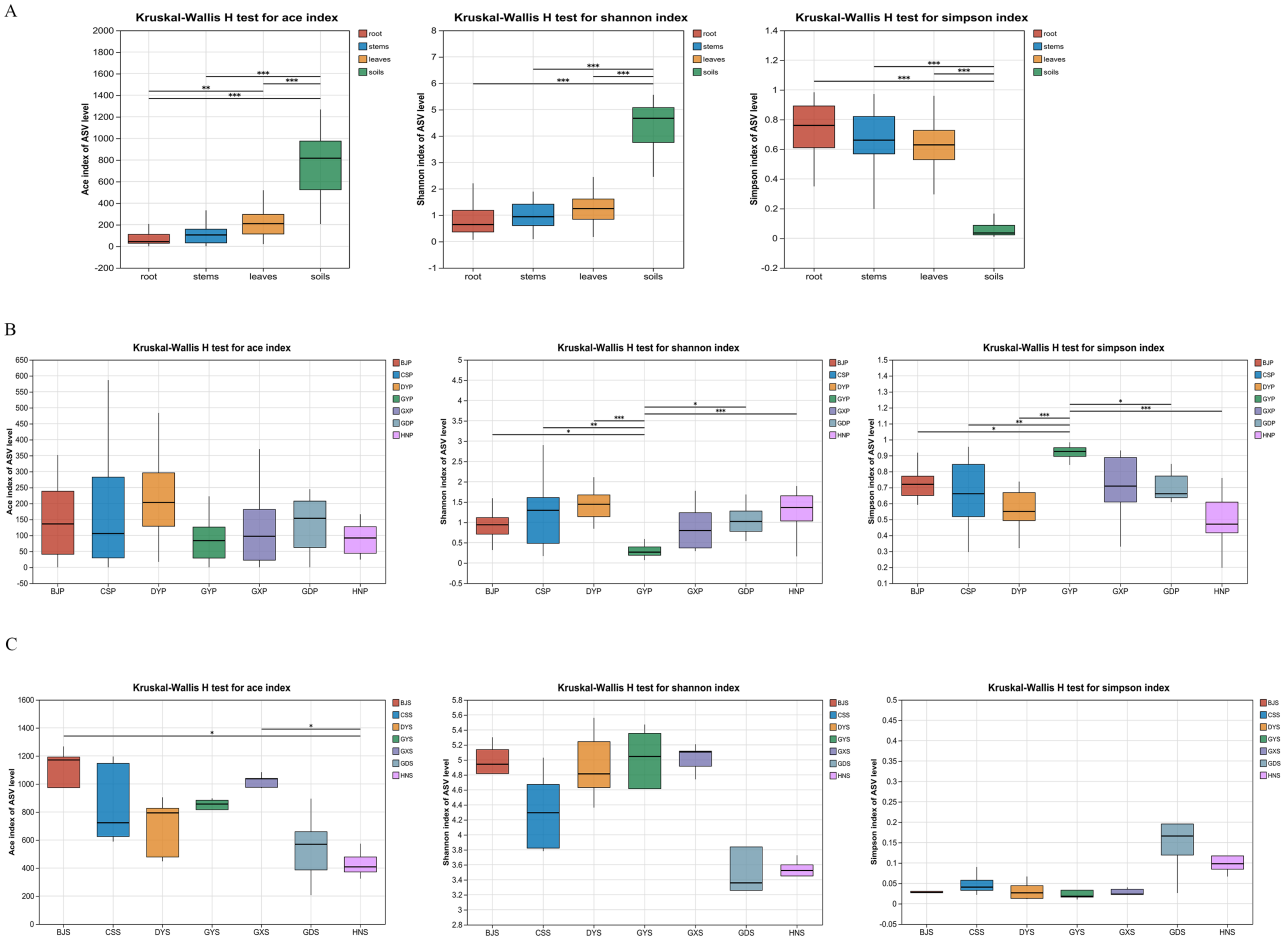
Alpha diversity indices of microbial communities from plants and rhizosphere soil from seven locations in China. **(A)** Analysis of Alpha diversity in different parts (leaves, stems, roots and soils); **(B)** Analysis of Alpha diversity in different region plant; **(C)** Analysis of Alpha diversity in different region soil.

The sparse curve tends to flatten out and the coverage reached 1.000, indicating that all samples are sufficient. In order to understand the changes of microbial community composition in samples from different geographical regions, endophytic fungi and rhizosphere soil microorganisms were analyzed using *α* diversity indices, including ACE, Shannon, and Simpson (Figure 3). The ACE and Shannon indices of soil communities were significantly higher than the corresponding indices of plant communities, and reflecting the greater richness of soil communities (Figure 3A; *p* < 0.05). On the contrary, The Simpson indices of plant communities were significantly higher than those of soil communities (Figure 3A; *p* < 0.05). The richness and diversity of microbial species were different among different sampling locations. For the plant communities, BJS, CSS and DYS had relatively high indices (Figure 3B; *p* < 0.05), while for soil, the three region also has the highest indices (Figure 3C; *p* < 0.05).

### 3.4 Analysis of microbial beta diversity

**Figure 4:**
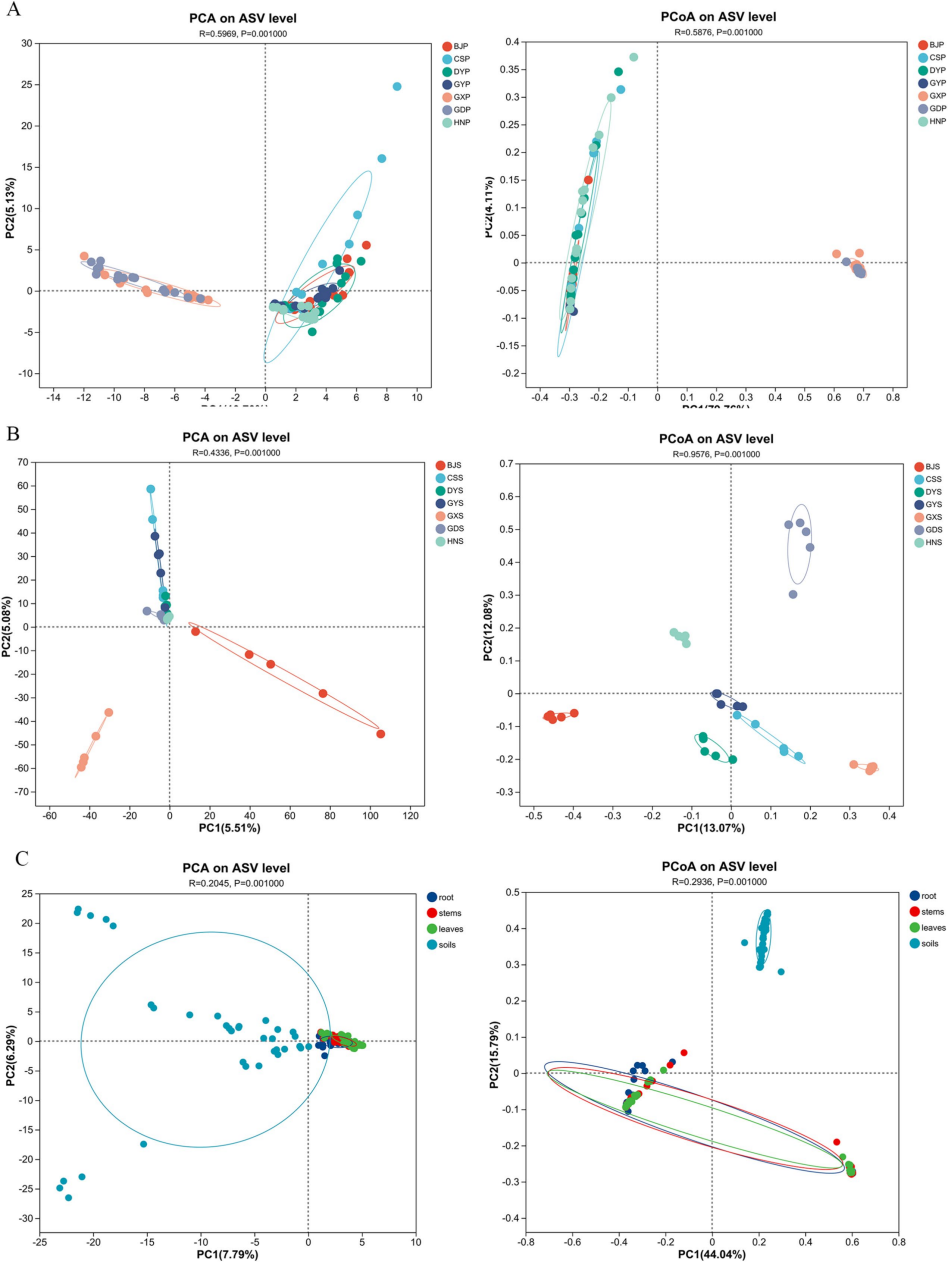
PCA and PCoA plots of microbial communities of plants and rhizosphere soil samples from seven regions. **(A)** PCA plot and PCoA plot of plant communities; **(B)** PCA plot and PCoA plot of rhizosphere soil communities; **(C)** PCA plot and PCoA plot of leaves, stems, roots and soils.

PCA analysis can reflect the difference and distance between samples by analyzing the community composition of different samples. The more similar the composition of sample species, the closer the distance reflected in PCA. PCoA analysis was conducted to assess the similarity of microbial community composition among samples based on weighted Unifrac distances. Based on the distance, the 105 plant samples were categorized into two groups, with GDP and GXP clustering together, and the remaining five samples forming another group (Figure 4A). PCoA analysis revealed that the 105 plant samples were categorized into seven distinct groups (Figure 4B), suggesting that the endophytic fungi in *A. crenata* Sims exhibited varied characteristics across different regions. Based on the Adonis analysis of the Bray-curtis distance matrix, the R2 value was 0.5876, indicating that there were significant differences among the seven plant communities, with statistical significance (Figure 4A; *p* < 0.01). The 35 rhizosphere soil samples were divided into three categories according to distance, among them, CSS, DYS, GYS, HNS and GXS samples are located in the first quadrant, GDS samples are distributed in the third quadrant, and BJS samples are distributed in the fourth quadrant (Figure 4B). The PCoA analysis showed that the 35 rhizosphere soil samples were divided into two categories (Figure 4B), whereas the differences within soil samples are highly similarity (*p* ≤ 0.01). The endophytic fungi varied significantly between plant and soil samples, were divided into two categories (Figure 4C).

### 3.5 Differential analysis of fungal community composition

**Figure 5:**
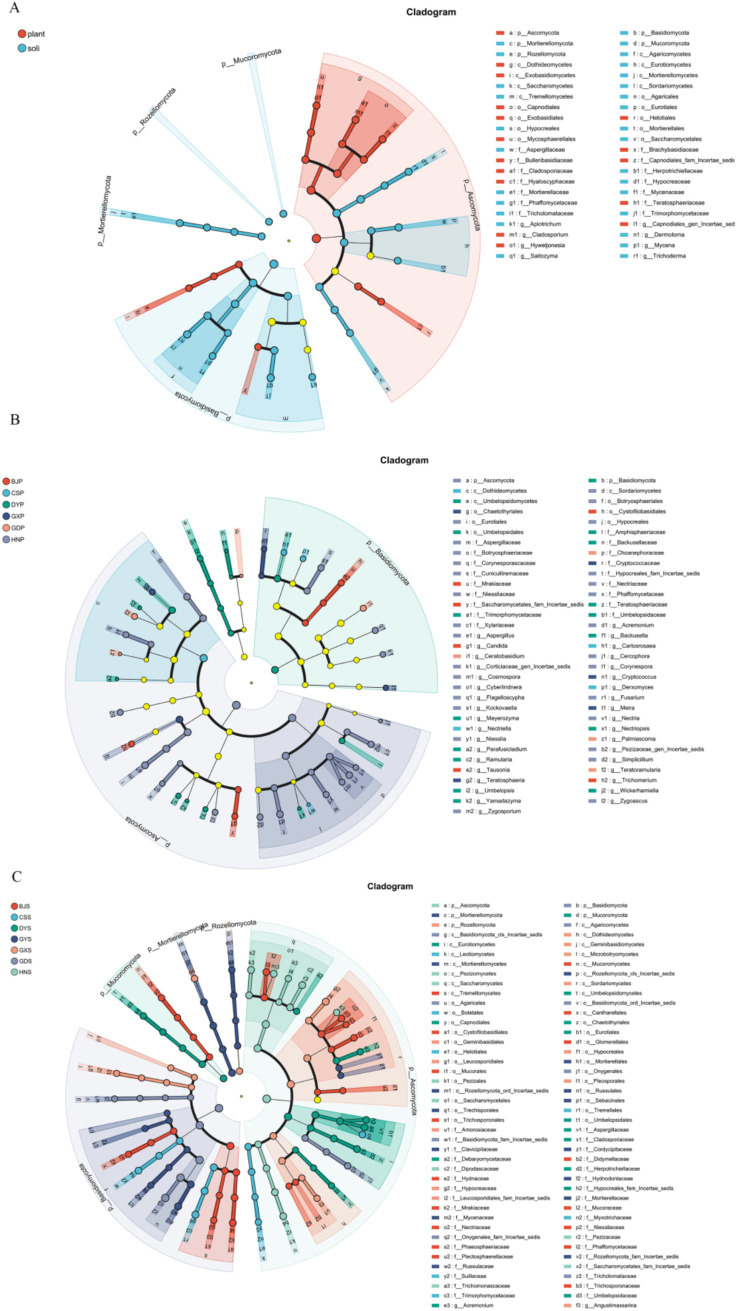
LEfSe cladograms display significantly abundant taxa in plants and soil. Taxa from various sites are depicted as colored dots. These taxonomic cladograms include only taxa that meet an LDA significance threshold of more than 3 for fungal communities. **(A)** Differences between endophytic fungi in plants and rhizosphere microorganisms; **(B)** Differences in endophytic fungi of plants in different regions; **(C)** Differences in rhizosphere soil microorganisms among different regions.

To further clarify the differences of fungal community structure in *A. crenata* Sims, LEfSe was used to analyze the different core fungi. Under the condition of an LDA threshold greater than 4, a total of 44 fungal differential indicator taxa were detected in the plant *vs* soli samples (Figure 5A). Some of the dominant phylum among these differential indicators include Ascomycota, Basidiomycota, Mortierellomycota, Mucoromycota, Rozellomycota. Under the condition of an LDA threshold greater than 3, a total of 80 fungal differential indicator taxa were detected in the plant samples in different region (Figure 5B). Some of the dominant genera among these differential indicators include *Aspergillus*, *Acremonium*, *Fusarium*, and so on. *Tausonia, Candida, and Trichomerium* emerged as core fungi in BJP, whereas *Carlosrosaea, Derxomyces, and Nectriella* were also notable in CSP; *Umbelopsis*, *Meyerozyma*, *Wickerhamiella*, *Yamadazyma*, *Backusella*, *Ramularia*, *Parafusicladium* in DYP; *Ceratobasidium*, *Teratoramularia*, *Palmiascoma* in GDS; *Teratosphaeria*, *Teratosphaeria*, *Meira* in GXP; *Aspergillus*, *Acremonium*, *Fusarium*, *Kockovaella*, *Zygosporium*, *Flagelloscypha*, *Niesslia*, *Simplicillium*, *Corynespora*, *Zygoascus*, *Cosmospora*, *Nectria*, *Cyberlindnera*, *Cercophora* in HNS. The taxonomic composition of fungal communities in *A. crenata* Sims from different regions was analyzed in detail, highlighting the differences of dominant phyla and genera in different growth environments.

LEfSe analysis was utilized to examine the variations in endophyte communities across diverse rhizosphere soil samples from various regions, revealing 124 differential indicator taxa in rhizosphere soil with an LDA threshold exceeding 4 (Figure 5C). At the genus level, *Tausonia*, *Barnettozyma*, *Fusarium*, *Eucasphaeria*, *Apiotrichum*, *Mucor*, *Minimedusa*, *Fusicolla* were dominant in BJS; *Saitozyma*, *Suillus*, *Phialomyces*, *Oidiodendron* in CSS; *Herpotrichiellaceae*, *Penicillium*, *Umbelopsis*, *Aspergillus*, *Cladosporium* in DYS; *Dermoloma*, *Onygenale*s and *Basidiomycota* in GDS; *Trichoderma*, *Podila*, *Angustimassarina*, *Leucosporidiales* in GXS; *Mycena*, *Russula*, *Rozellomycota* in GYS; *Candida*, *Pezizaceae*, *Zygoascus*, *Nectria* in HNS. These indicators exhibited distinct variations across different regions of rhizosphere soil samples. This detailed analysis provides insights into the taxonomic composition of fungal communities in *A. crenata* Sims and the associated soil, highlighting differences in dominant phyla and genera between the two environments.

### 3.6 Microbial co-occurrence network analysis

**Figure 6:**
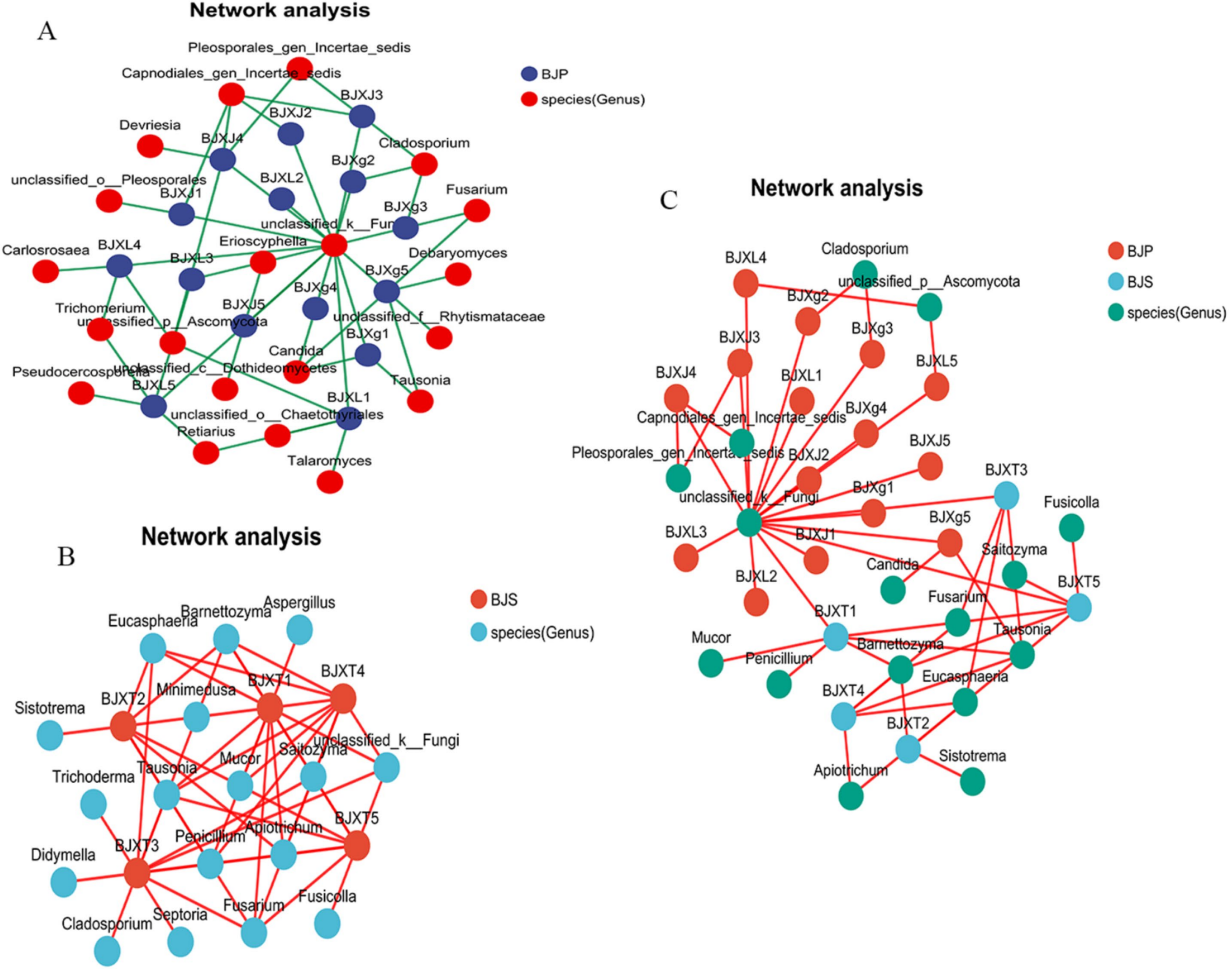
Co-occurrence network analysis of fungi in plants **(A)**, soil **(B)** and plant–soil **(C)** at the genus level.

To compare the co-occurrence patterns of *A. crenata* Sims plant fungal communities, this study selected the BJ plant samples and their corresponding BJ soil samples to construct co-occurrence networks. The network properties revealed that BJS had more nodes, edges, average neighbors, network heterogeneity, and network centralization than BJP (Table 3). This indicates that the soil network possesses a higher degree of natural connectivity in comparison to the plant network, thereby suggesting that interactions among soil microbial communities may be stronger than those within plant communities. Key genera identified, included *Cladosporium*, *Tausonia*, *Erioscyphella*, *Candida*, *Trichomerium*, *Devriesia*, *Fusarium*, *Retiarius* in the *A. crenata* Sims (Figure 6A). In the soil network, key genera included *Tausonia*, *Sistotrema*, *Eucasphaeria*, *Barnettozyma*, *Fusarium*, *Mucor*, *Barnettozyma*, *Penicillium*, *Fusicolla*, *Apiotrichum*, etc. (Figure 6B). When considering all shared microorganisms in plant and soil samples, the key genera identified encompassed *Tausonia, Apiotrichum*, *Barnettozyma*, *Fusarium*, *Penicillium*, *Saitozyma*, *Trichoderma*, and so on (Figure 6C). The results suggest that interactions within soil fungal communities are closely linked, indicating that plant and soil communities harbor dominant and closely interacting microbiota.

**Table 3:** Network property statistics for endophytic fungi networks in plant (BJP) and soil (BJS).

Sample	Number of nodes	Number of edges	Average neighbors	Network density	Network heterogeneity	Network centralization
BJP	385	909	4.722	0.012	3.093	0.331
BJS	706	1843	5.221	0.007	5.969	0.634

### 3.7 Fungal gene function prediction

**Figure 7:**
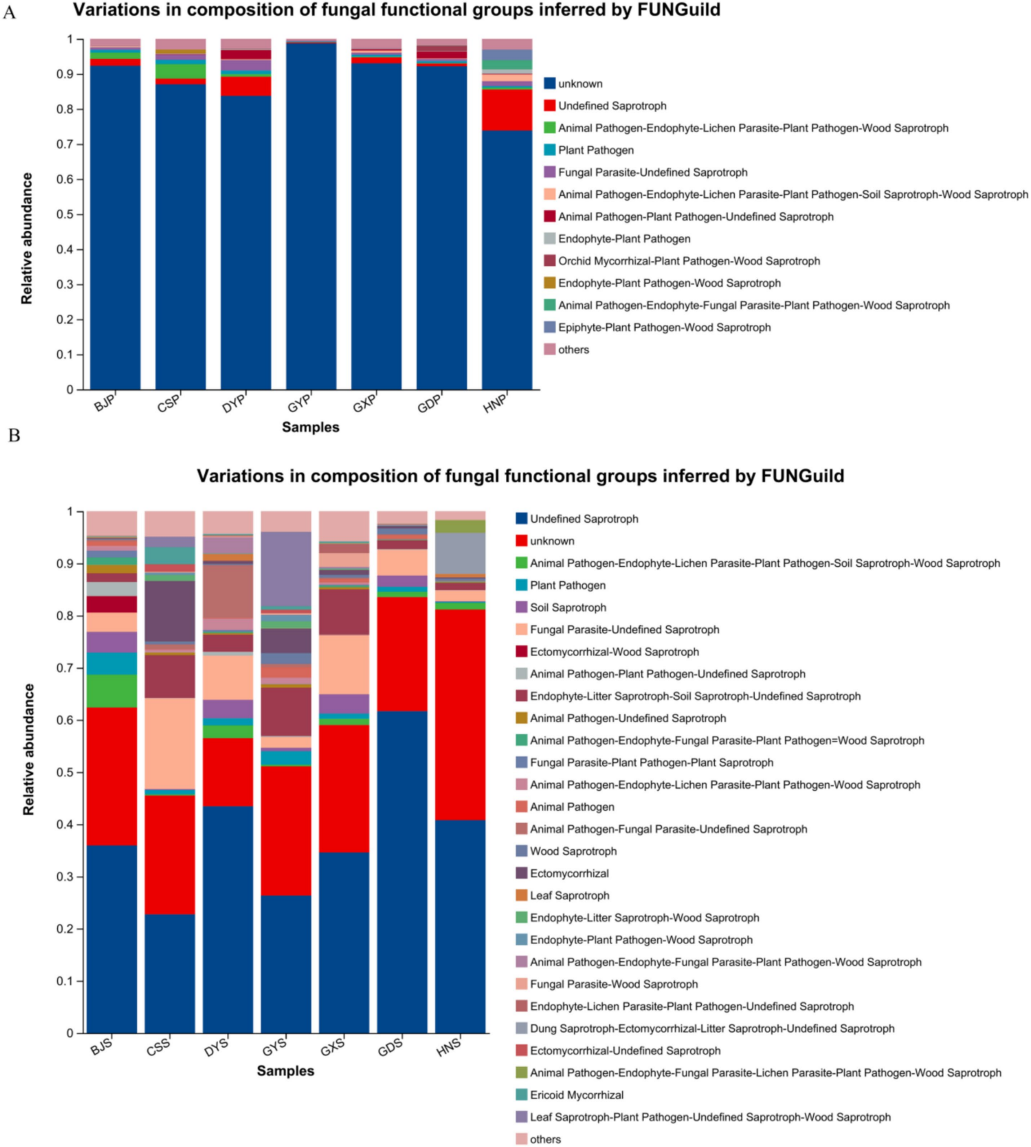
Relative abundance chart of fungal gene function predictions. **(A)** Relative abundance of fungal gene function predictions in plants. **(B)** Relative abundance of fungal gene function predictions in soil.

Functional predictions of fungal taxa in both soil and plants were conducted using FunGuild. The results indicated that the primary metabolic types in plants comprised Undefined Saprotroph, Plant Pathogen, Fungal Parasite, Animal Pathogen, Endophyte-Plant Pathogen, among others (Figure 7A). The Undefined Saprotroph, Animal Pathogen, Plant Pathogen, Soil Saprotroph, Fungal Parasite, Wood Saprotroph, Leaf Saprotroph were found in the soil (Figure 7B). These shared metabolic types were similar between plants and soil, and dominant genera in both plant and soil communities are associated with each other. But, the soil endophytic fungi communities are more active, and may be stronger than those within plant communities.

### 3.8 Relationships between rhizosphere soil microorganisms and soil physiochemistry, active ingredient of root

**Figure 8:**
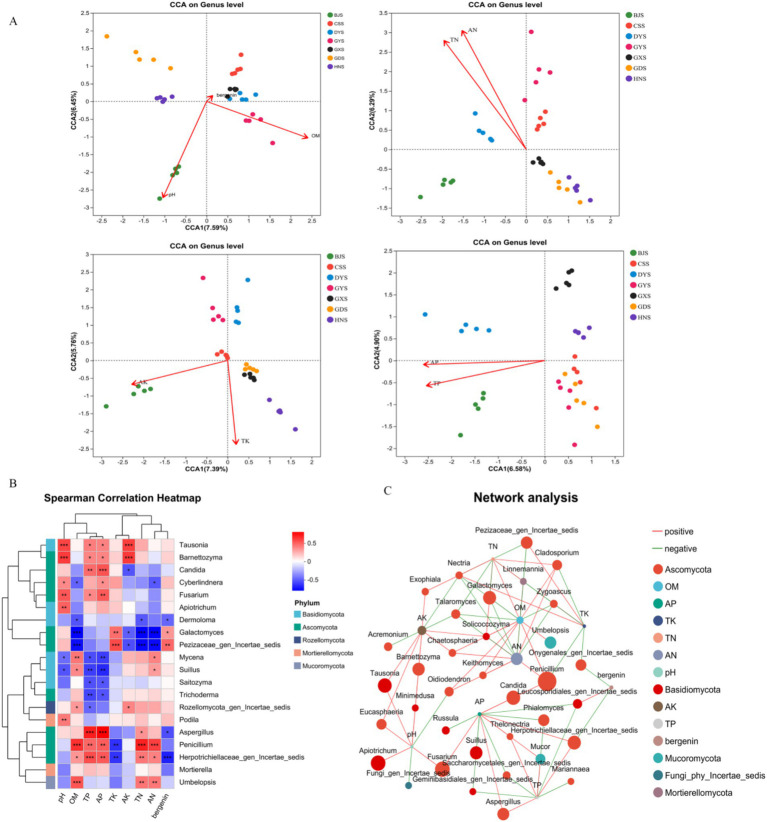
Analysis of correlations between soil physicochemical properties, active ingredients, and dominant soil microorganisms. **(A)** Canonical correspondence analysis (CCA); **(B)** Spearman Correlation Heatmap; **(C)** Network analysis.

Examining the relationships between physicochemical properties, soil microbial communities, and active compounds is crucial for maintaining the quality of *A. crenata* Sims medicinal herbs. The CCA analysis indicated that the fungal community composition in the rhizosphere soil of BJ was positively correlated with pH, AK, AP, and TP in the rhizosphere soil, with a more pronounced correlation with AK (Figure 8A). For DY, the fungal community composition was positively correlated with OM, TN, AN, TP, and AP, with a more pronounced correlation with TP and AP (Figure 8A). In the rhizosphere soil of GD, GX, and HN, the fungal community composition was positively correlated with TK (Figure 8A). The active ingredient bergenin in the root was positively correlated with GX (Figure 8A). CCA can reveal the correlations between species distribution and environmental factors, but it cannot directly explain the interactions among species. So, Spearman’s correlation heatmap was used to analyse the relationships between the rhizosphere soil fungal genera and soil physiochemistry and active ingredient.

The content of TN and AN exhibited a highly significant positive correlation with the genera *Mycena*, *Penicillium*, and *Suillus*, whereas a highly significant negative correlation was observed with *Cyberlindnera*, *Dermoloma*, and *Galactomyces* (Figure 8B). There was a highly significant positive correlation between the TK content and the genera *Cyberlindnera*, *Galactomyces*, and a highly significant negative correlation with *Penicillium* (Figure 8B). AK were significantly positively correlated with the *Barnettozyma* and *Tausonia*, and highly significantly negatively correlated with *Candida* and *Galactomyces* (Figure 8B). In addition, OM were significantly positively correlated with the *Suillus*, *Mycena*, *Penicillium*, and highly significantly negatively correlated with *Galactomyces*, *Dermoloma*, *Cyberlindnera* (Figure 8B). We also found that TP and AP were significantly positively correlated with the genera *Aspergillus*, *Barnettozyma*, *Candida*, *Cyberlindnera*, *Fusarium*, *Penicillium*, and *Tausonia*, and exhibited highly significant negative correlations with *Mycena*, *Saitozyma*, *Suillus*, and *Trichoderma* (Figure 8B). There was a highly significant positive correlation between the content of bergenin and the bacterial genera *Galactomyces*, and a highly significant negative correlation with *Aspergillus* (Figure 8B). To some degree, the makeup of the rhizosphere fungi community influences the production of active constituents within *A. crenata* Sims. The factors pH, OM, TN, AN, TP, AP, TK, and AK were associated with Ascomycota, Basidiomycota, Mucoromycota, Rozellomycota (Figure 8C). In summary, soil physicochemical factors influence the alterations in fungal community composition within the rhizosphere soil, with TP and AP contents being particularly closely correlated with these changes.

## 4 Discussion

Utilizing high-throughput sequencing technology, this study is the first to explore the diversity, community structure, and composition of endophytic fungi and rhizosphere soil microbial communities in *Ardisia crenata* Sims. The diversity of endophytic fungi was highest in soil, followed by leaves. Studies have shown that the survival of endophytic fungi within plants is highly dependent on the resources provided by their host plants, whereas the resource acquisition pathways of soil endophytic fungi are more diverse (Hardoim et al., 2015). Meanwhile, the diversity and abundance of endophytic fungi within plants are limited by the host plant species, growth stage, and environmental conditions, whereas the ecological niche of endophytic fungi in soil is broader (Compant et al., 2019). In short, soil endophytic fungi have wider ecological niche, more flexible resource acquisition, more efficient propagation, etc., which makes soil endophytic fungi far more abundant and diverse than plant endophytic fungi. Leaves exposed to the air can harbor more fungal spores, which may produce rich carbohydrates through photosynthesis, potentially explaining the abundance of endophytic fungi in leaves. We have discovered a large number of unidentified endophytic fungi within the roots, stems, and leaves of plants, finding that warrants further research. Therefore, we will also conduct the isolation and identification of endophytic fungi from *A. crenata* Sims using traditional culturing techniques.

The results on community composition revealed that Ascomycota, Basidiomycota, and Mortierellomycota were the predominant fungal phyla in both the plant and soil samples, consistent with numerous previous reports highlighting Ascomycota and Basidiomycota as dominant groups across a broad array of plant endophytic and rhizosphere soil fungi (Karthikeyan et al., 2008; Egidi et al., 2019; Tang et al., 2024). Our research has showed that the Ascomycota are diverse in the soil, studies have indicated that Ascomycota plays a crucial role in increasing nutrient in the soil (Voíková and Baldrian, 2013). Fungal function prediction revealed that Undefined Saprotroph, Plant Pathogen, Fungal Parasite, Animal Pathogen, Endophyte-Plant Pathogen, Soil Saprotroph, Wood Saprotroph, Leaf Saprotroph were present in greater proportions in the these region. Due to the presence of the fungal parasite *Trichoderma* in these samples, it is important to note that *Trichoderma* serves as a crucial biocontrol agent, exhibiting antagonistic effects against numerous soil-borne pathogens (Gurr et al., 2000; Whipps, 2001). These pathways may contribute to plant growth, development, and the production of bioactive compounds, while soil microbial communities also engage in metabolic pathways associated with soil health and the decomposition of organic matter.

The Alpha diversity index found variations in the microbial community at different sampling points, with the highest fungal diversity in plant sample DY and CS, while the highest fungal diversity in soil was in BJ and GX. Thus, there are notable disparities in microbial communities and species compositions between plant and soil samples from various regions. These variations can be attributed to the influence of environmental factors, including pH, humidity, temperature, oxygen content, and soil properties across different geographical regions. Previous studies have demonstrated that microbial diversity can be affected by various environmental factors, including temperature, humidity, oxygen content, and soil properties across different regions (Zheng et al., 2023). The rhizosphere microbial occupy a wider range of ecological niches, including decomposing organic matter, forming symbiotic relationships with plants, and acting as pathogens (Bardgett and Putten, 2014). Furthermore, endophytic fungi perform multiple functions in soil, such as decomposing complex organic substances like lignin and cellulose, and participating in nutrient cycling, among others (Zanne et al., 2020). The diversity of these functions supports a higher species diversity. The greater the abundance of endophytic fungi species, the more it may promote the synthesis and accumulation of active components in host plants. Therefore, unraveling the involvement of soil variables in the regulatory mechanism of secondary metabolite and related microbial diversity in *A. crenata* Sims is crucial to optimize plant–soil interaction in *A. crenata* Sims plants cultivation.

We have discovered that *A. crenata* Sims primarily grows in subtropical monsoon climate regions at altitudes ranging from 1,000 to 2,000 meters. It prefers to grow in moist forest underbrush and often coexists with pine needles. Different symbiotic environments may also lead to differences in endophytic fungi. So, we analyzed the physicochemical properties of the soil, such as pH value, organic matter, total nitrogen, among others, exert an influence on the population structure and function of rhizosphere microorganisms. The CCA analysis reveals differences in pH, organic matter, total nitrogen (TN), available nitrogen (AN), total potassium (TK), available potassium (AK), total phosphorus (TP), and available phosphorus (AP) content among the areas in Guizhou, Hunan, Guangxi, and Guangdong. Acidic soil may be more conducive to the growth of acid-tolerant fungi, while alkaline soil may promote the reproduction of other groups. Different environmental conditions, such as soil pH and nutrient content, may shape unique fungal communities through environmental filtering (Tedersoo et al., 2014). The pH level, available nutrients, and trace elements in the soil all impact the growth and development of medicinal plants, and can even affect the content of active compounds (Wu et al., 2013). Nutrient elements, including carbon (C), nitrogen (N), phosphorus (P), and potassium (K), undergo leaching and degradation from plant residues, subsequently penetrating into soil reserves. These elements are then absorbed and utilized by plants, contributing to the formation of nutrient cycles. The C:N:P ratio, a key indicator of litter quality, significantly influences microbial community structure, as evidenced by studies showing that phosphorus addition can alter these ratios and subsequently impact microbial composition (Zheng et al., 2018). The differences in the quality and quantity of plant residues, root exudates and organic matter may affect the composition of fungal communities. The plants rich in cellulose may promote the growth of cellulose-degrading fungi, while those rich in protein may be more conducive to the reproduction of saprophytic fungi (Baldrian, 2017). These differences exhibit varying degrees of correlation with the root zone soil community structure.

Through prolonged adaptation to their growth environment, medicinal plants internal environment regulates the accumulation of secondary metabolites (Guo et al., 2013). An analysis of the correlations among the physicochemical properties, soil microbes, and active compounds of *A. crenata* Sims revealed that *Aspergillus*, *Barnettozyma*, *Cyberlindnera*, *Candida*, *Dermoloma*, *Fusarium*, *Galactomyces*, *Penicillium*, *Mycena*, *Tausonia*, *Saitozyma*, *Suillus*, and *Trichoderma* were either positively or negatively correlated with soil physicochemistry or active compounds. *Aspergillus* is a saprophytic fungus, and plays a crucial role in decomposing organic substances, including plant residues, cellulose, lignin and chitin, etc. Through decomposing complex organic compounds, it releases carbon, nitrogen, and other nutrients into the soil, promoting nutrient cycling and supporting plant growth. The genus *Fusarium* is a group of filamentous fungi that are widely distributed in soil and plant environments, and can promote nutrient cycling by decomposing complex organic compounds such as cellulose and lignin, and release carbon and nitrogen into the soil. *Fusarium* can establish a beneficial interaction with the host, playing a role in biological control and promoting plant growth, and has the potential to be developed as a biocontrol agent. *Trichoderma* and *Penicillium* are considered to be the ecological groups to grow on the root surface. *Dermoloma,* a genus of basidiomycete fungi within the Tricholomataceae family, is instrumental in the decomposition of organic matter and the facilitation of nutrient cycling within its ecosystem (Sánchez-García et al., 2021). *Penicillium* exhibits the capacity to decompose organic matter, playing a pivotal role in the natural material cycle and thus contributing to both environmental protection and the augmentation of soil fertility*. Tausonia* plays a crucial role in the soil microbial community, aiding in the maintenance of soil health and ecological balance (Lu et al., 2024). Endophytic fungi form symbiotic relationships with plants, exchanging nutrients and signaling molecules with them, thereby influencing the chemical composition of plants. Overall, these are important for improving the quality and yield of *A. crenata* Sims.

In a comparative analysis of seven samples, GX exhibited the highest bergenin content, with HN, BJ, CS, GY, and GD following in descending order, while DY had the least bergenin content. At a certain, the composition of the rhizosphere fungi community affects the synthesis of active components in *A. crenata* Sims. Nitrogen, phosphorus, and potassium, among other elements, can influence the synthesis and metabolism of flavonoids in medicinal plants by modulating carbon-nitrogen metabolism, the metabolism of endogenous plant hormones, and the activity of key enzymes. The interaction between fungi and plants may regulate the synthesis of secondary metabolites in plants. Some endophytic fungi colonize within plant tissues and promote the synthesis of active compounds by producing metabolites or activating the plant’s defense responses. *Aspergillus* produces various secondary metabolites, including alkaloid, phenolics, flavonoids, terpenes, steroids, etc. (Shlyk et al., 2025; Gupta et al., 2025). Nowdays, as many as 185 antimicrobial natural products as secondary metabolites had been discovered from *Fusarium*, and having antibacterial, anti-Gram-Positive Bacterial, anti-Gram-Negative Bacterial, antifungal, antiviral, antiparasitic, etc. (Xu et al., 2023; Li et al., 2020). *Fusarium* also significantly enhances the content of flavonoids by activating plant defense responses and metabolic pathways (Zhong et al., 2016). *Penicillium* is a high-yielding strain for secondary metabolites, containing polyketides, tetrameric acids, phenols and alkaloids (Le et al., 2019). Beneficial endophytic fungi, such as *Penicillium* and *Fusarium*, can enhance the yield and quality of medicinal materials by exerting various ecological functions (Li et al., 2024). Therefore, endophytic fungi may be closely related to the accumulation of chemical components through various mechanisms, such as producing secondary metabolites, regulating plant metabolic pathways, and participating in organic matter decomposition.

This study also has some limitations: (1) The repeatability of plant and rhizosphere soil samples from the same location may not have accounted for the potential parent-plant relationships. (2) The single point in time may not capture the seasonal or temporal variations of fungal and plant communities, and it cannot represent all environmental conditions and ecosystem types. These factors may affect the composition of microbial communities and introduce result deviations. In the future, we will (1) the sample size will be increased and the repetition strategy will be improved to consider spatial and genetic relationships; (2) the geographical coverage will be expanded to cover a wider range of ecosystems and environmental conditions; (3) more environmental variables and time sampling will be included to better understand their effects on microbial and plant communities; (4) comparing microbial communities in cultivated and wild cinnabar roots is valuable.

## 5 Conclusion

This study pioneers the use of high-throughput sequencing to investigate the fungal community structure within the leaves, stems, roots, and rhizosphere soil of *A. crenata* Sims across seven diverse regions in China. There are significant differences in the composition of endophytic fungal communities and rhizosphere soil fungal communities, and rhizosphere soil fungi are abundant in endophytic fungi of *A. crenata* Sims. However, there are a large number of unidentified species in the endophytic fungi of *A. crenata* Sims. The diversity of the rhizosphere fungal community has a certain correlation with soil physicochemical properties and bergenin in *A. crenata* Sims, *Aspergillus*, *Fusarium*, *Penicillium*, *Tausonia* and *Trichoderma* are the key fungal genera. This study provides microbial regulation strategies for targeted cultivation of *A. crenata* Sims, suggesting that optimizing soil phosphorus content can increase bergenin production.

## Data Availability

The original contributions presented in the study are publicly available. This data can be found at: PRJNA1231709.
